# Artificial Intelligence and Radiomics in Cholangiocarcinoma: A Comprehensive Review

**DOI:** 10.3390/diagnostics15020148

**Published:** 2025-01-10

**Authors:** Marta Zerunian, Tiziano Polidori, Federica Palmeri, Stefano Nardacci, Antonella Del Gaudio, Benedetta Masci, Giuseppe Tremamunno, Michela Polici, Domenico De Santis, Francesco Pucciarelli, Andrea Laghi, Damiano Caruso

**Affiliations:** 1 Department of Medical Surgical Sciences and Translational Medicine, Sapienza–University of Rome, Radiology Unit–Sant’Andrea University Hospital, 00189 Rome, Italy; tiziano.polidori@uniroma1.it (T.P.); federica.palmeri@uniroma1.it (F.P.); stefano.nardacci@uniroma1.it (S.N.); antonella.delgaudio@uniroma1.it (A.D.G.); benedetta.masci@uniroma1.it (B.M.); giuseppe.tremamunno@uniroma1.it (G.T.); michela.polici@uniroma1.it (M.P.); domenico.desantis@uniroma1.it (D.D.S.); francesco.pucciarelli@uniroma1.it (F.P.); andrea.laghi@uniroma1.it (A.L.); damiano.caruso@uniroma1.it (D.C.); 2 PhD School in Translational Medicine and Oncology, Department of Medical and Surgical Sciences and Translational Medicine, Faculty of Medicine and Psychology, Sapienza University of Rome, 00189 Rome, Italy

**Keywords:** cholangiocarcinoma, radiomics, artificial intelligence, deep learning, ultrasonography, computed tomography, magnetic resonance imaging

## Abstract

Cholangiocarcinoma (CCA) is a malignant biliary system tumor and the second most common primary hepatic neoplasm, following hepatocellular carcinoma. CCA still has an extremely high unfavorable prognosis, regardless of type and location, and complete surgical resection remains the only curative therapeutic option; however, due to the underhanded onset and rapid progression of CCA, most patients present with advanced stages at first diagnosis, with only 30 to 60% of CCA patients eligible for surgery. Recent innovations in medical imaging combined with the use of radiomics and artificial intelligence (AI) can lead to improvements in the early detection, characterization, and pre-treatment staging of these tumors, guiding clinicians to make personalized therapeutic strategies. The aim of this review is to provide an overview of how radiological features of CCA can be analyzed through radiomics and with the help of AI for many different purposes, such as differential diagnosis, the prediction of lymph node metastasis, the defining of prognostic groups, and the prediction of early recurrence. The combination of radiomics with AI has immense potential. Still, its effectiveness in practice is yet to be validated by prospective multicentric studies that would allow for the development of standardized radiomics models.

## 1. Introduction

Cholangiocarcinoma (CCA) is a malignant disease that originates from cells of the biliary tract [1]. CCA accounts for approximately 3% of all gastrointestinal tract neoplasms [2], and it is the second most common liver tumor (15–20%) after hepatocellular carcinoma (HCC) [1,3]. Regarding its pathogenesis, several risk factors are related to CCA onset, such as primary sclerosing cholangitis, liver cirrhosis, liver fluke infections, inflammatory bowel disease, toxins, and metabolic conditions [4].

The anatomical location of the primary tumor divides the CCA into intrahepatic (iCCA) and extrahepatic (eCCA) variants. In the eCCA variant, perihilar (pCCA) and distal (dCCA) sub-types can be identified based on their location in the bile duct [5]. pCCA is the most common variant of CCA, accounting for about 60% of all CCAs, and originates from the right or left hepatic duct or, alternatively, from their junction [2,4,6]. iCCA originates from the second-order bile ducts, up to the junction of the cystic duct with the common bile duct, and accounts for about 10% of all CCAs [2,4,6]; dCCA affects the common bile duct from the insertion of the cystic duct to the ampulla of Vater and accounts for 20–30% of all CCAs [2,4].

The literature describes a rare type of hepatic malignancy showing the clinicopathological and radiological features of CCA together with the features of hepatocellular carcinoma (HCC), named combined hepatocellular-cholangiocarcinoma (CHC). CHC has prevalence of 0.4–1.4% approximately [2,7], and shows aggressive behavioral characteristics; consequently, the prognosis of CHC is poor, with a 5-year survival rate of 17.7% [7]. CCA usually does not present clinical symptoms at early stages as they tend to appear when the disease is already advanced, and these symptoms include jaundice, vomiting, and a rise of cholestasis indices [8].

CA 19-9 (Carbohydrate Antigen 19–9) is the most widely used a biological marker in CCA, and although it tends to be high in other cholangiopathies as well, it allows for a differential diagnosis between HCC and CCA [9]. Furthermore, it is also helpful during the follow-up period to evaluate the response to treatment and signs of recurrence after surgical resection [8].

The diagnosis of CCA is challenging due to its anatomical location and late presentation [8]; therefore, it requires a multidisciplinary approach based on clinical presentation, imaging, and biopsy [9]. In this context, radiological evaluation plays a central role but almost always requires histopathological confirmation through percutaneous biopsy or through invasive procedures such as endoscopic retrograde cholangiopancreatography (ERCP) or percutaneous transhepatic cholangiography (PTC) [8,9].

In the management of CCA, surgical resection remains the only curative therapy. However, only a few patients are able to receive surgery since about 70% of patients present with advanced-staged CCA, and surgery is not recommended as a curative treatment; thus, palliative care remains the only treatment option [10]. The first line of palliative care treatment involves the use of a chemotherapy regimen based on cisplatin and gemcitabine [10], with confirmed benefits in terms of survival [9]. However, other therapeutic options include non-surgical approaches, such as radiofrequency ablation and transarterial chemoembolization (TACE) [9].

Patients with unresectable pCCA smaller than 3 cm in size and non-distant spreading are candidates for liver transplantation, which shows better results when associated with neoadjuvant chemoradiotherapy [11].

The prognosis for CCA is very poor, with a 5-year survival rate of less than 5% in iCCA and 17% in eCCA; surgery improves the prognosis of patients with iCCA, with a 5-year life expectancy of 20–44% [8]. Unfortunately, CCA diagnosis is usually belated, considerably affecting the management and prognosis of these patients.

Nowadays, radiomics and artificial intelligence (AI) are emerging as new diagnostic tools in oncology [12,13]. Among the various AI methodologies used in the medical field, machine learning (ML) and deep learning (DL) allow for improvements in the efficiency of the care process [14]. In this context, radiomics also proves to be a promising non-invasive tool in the management of the clinical decision-making process through the evaluation of numerous quantitative features extracted from radiological images [15].

Artificial intelligence has proved to be a valuable tool in supporting the diagnosis of CCA through integrating serological and histological stains with imaging data, as well as providing prognostic estimates for potential treatment options [14]. Building on this, our review aims to provide a comprehensive, yet accessible overview of the current imaging modalities used in clinical practice for CCA diagnosis, with a particular focus on the role of AI and radiomics in the diagnostic–therapeutic management of these patients. Specifically designed for beginners and readers not yet familiar with these emerging technologies, it offers a clear and easy-to-understand summary of the state of the art, illustrating how AI and radiomics are applied across different imaging modalities, the unique advantages they offer depending on the modality, and their potential to enhance diagnostic accuracy and treatment planning. Furthermore, we critically examine the limitations of these technologies to provide readers with a balanced perspective on both their strengths and the challenges they currently face.

## 2. Imaging Modalities

Diagnosing CCA remains a significant challenge in cross-sectional imaging due to the tumor’s tendency to present incidentally and at an advanced stage, often as a result of nonspecific or absent clinical symptoms. Given these difficulties, an accurate diagnosis of CCA requires a multidisciplinary approach where cross-sectional imaging plays a central role [8,9]. These imaging techniques not only help to detect the presence of a lesion but also provide crucial information regarding its extent, vascular involvement, and potential distant metastases [16,17]. The diagnostic process typically begins with ultrasound (US) as a first-line examination, followed by computed tomography (CT) and magnetic resonance imaging (MRI), which work together to offer a more detailed and complementary evaluation of the tumor, ensuring thorough and precise staging evaluation [16,18].

Morphologically, CCA is divided, according to the Liver Cancer Study Group of Japan, into three growth patterns: mass-forming, periductal-infiltrating, and intraductal growth-types. This morphological classification allows for the prediction of the stage of evolution of the tumor and its development, and helps in the treatment decision process [18,19]. Both iCCA and eCCA can exhibit these growth patterns; however, iCCA’s most common growth type is the mass-forming type, while eCCA occurs more frequently in the remaining two types [16].

Another important classification concerns pCCA, which, according to the Bismuth–Corlette classification (Table 1), is divided into four types based on its hilar longitudinal extension [20].

With regards to the staging of iCCA and pCCA, imaging techniques, in particular CT, can identify vascular involvement and extrahepatic invasion, while in staging eCCA, these methods are not always able to determine the spread of the tumor compared to the bile duct. However, new MRI techniques allow for better tissue characterization, making this the method of choice for diagnosis and staging according to current guidelines [21].

Imaging plays an essential role not only in the diagnosis and treatment planning for CCA but also in post-therapy surveillance, ensuring comprehensive management of the disease. In follow-up care, computed tomography (CT) is the preferred method for detecting biliopathy, while magnetic resonance imaging (MRI) with diffusion sequences proves particularly valuable for assessing disease persistence or recurrence after surgery. These imaging techniques collectively support clinicians in diagnosing CCA, selecting the most appropriate treatment strategies, and monitoring patients during post-treatment surveillance to ensure the timely detection of complications or recurrence [22].

### 2.1. Ultrasonography (US)

US is the first non-invasive imaging method performed in patients with suspected liver or biliary tract disease due to its accessibility and ease of use [12]. While US is not always capable of directly identifying CCA, it can reveal indirect signs of bile duct injury that may raise suspicion for malignancy [23]. Its diagnostic accuracy for malignancy is relatively low, at approximately 37%, and depends on factors such as the disease’s location and the operator’s expertise [22]. Nevertheless, US demonstrates a sensitivity of 55–95% and a specificity of 71–96% in detecting the site of bile duct strictures, making it a valuable initial tool in the diagnostic pathway [24].

**Figure 1:**
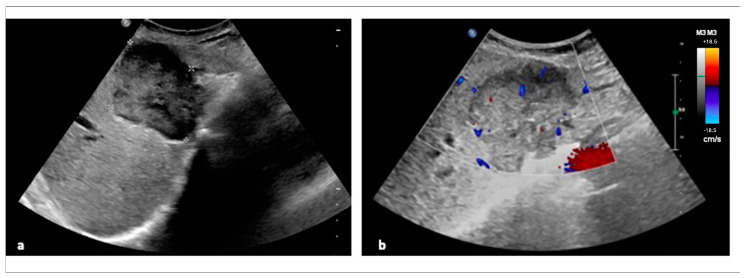
Ultrasound features of iCCA. Ultrasound features of iCCA in a 79-year-old female: (**a**) a hypoechoic mass with polylobed margins occupying the IV hepatic segment. (**b**) Signs of perilobular and intranodular vascularization on Color Doppler examination. Abbreviation: iCCA, intrahepatic cholangiocarcinoma.

The appearance of radiological images depends on the morphological classification of the type of CCA: mass-forming CCA appears as a homogeneous mass with a peripheral hypoechoic profile (Figure 1), with the expression of healthy hepatic parenchyma compressed by tumor cells; periductal-infiltrating CCA appears as a small mass or thickening of the bile duct with or without dilatation of the bile ducts; while intraductal growing-type CCA appears as a dilation of the biliary tract caused by an intraductal polypoid lesion, which may also be absent [25]. 

The ultrasound characteristics of iCCA are influenced by factors such as the presence of necrosis, the amount of mucin and fibrous tissue, and the size of the mass: lesions greater than 3 cm are hyperechoic, while lesions smaller than 3 cm are generally hypoechoic [26]. The sonographic appearance of iCCA is similar to HCC, but can be distinguished by assessing portal branch involvement because iCCA less frequently causes portal branch thrombosis [27].

Contrast-enhanced ultrasound (CEUS) can be used in the diagnosis of iCCA as it allows for the evaluation of the vascular pattern of the lesion. In most cases, iCCa shows a peripheral rim-like enhancement in the arterial phase and an early wash-out, usually within 60 s. CEUS can also help distinguish between iCCA and HCC; the latter exhibits homogeneous enhancement in the arterial phase and delayed washout [28].

A diagnosis of pCCa may be suspected when intrahepatic bile duct dilatation is recognizable upon ultrasound examination, with an abrupt discontinuation near the bifurcation of the hepatic duct [29].

dCCA is manifested by the dilatation of the intra- and extrahepatic bile ducts, with characteristic Color Doppler images showing the blood flow signal infiltrating the bile duct wall [29,30].

### 2.2. Computed Tomography (CT)

Contrast-enhanced CT (CECT) is the most commonly used imaging method for diagnosing CCA [27], with a detection rate of 94–100% [29]. Its diagnostic accuracy varies depending on the type of CCA, reflecting its strengths and limitations in specific scenarios. For intrahepatic cholangiocarcinoma (iCCA), CECT achieves an accuracy of 70%, with sensitivity and specificity values of 78% and 80%, respectively [27]; in cases of extrahepatic cholangiocarcinoma (eCCA), CECT demonstrates a diagnostic accuracy ranging from 78.6% to 92.3%, ref. [31], and particularly excelling in diagnosing perihilar cholangiocarcinoma (pCCA), where it achieves an accuracy of 92% and a sensitivity of 60% [24]. These figures underline the critical role of CECT in diagnosing and characterizing different forms of CCA.

CT allows for the identification of the neoplasm in order to evaluate the extension to the adjacent tissues [21] and to detect lymph node involvement, the presence of metastases [21], and the possible involvement of the hepatic artery and the portal vein [16].

Commonly, the CECT protocol provides an examination of an arterial phase, acquired 15–18 s after intravenous injection of a contrast agent with the bolus tracking technique; as well as an examination of the venous portal phase, acquired 60–65 s after injection; and a delayed phase between 3 and 5 min [32].

**Figure 2:**
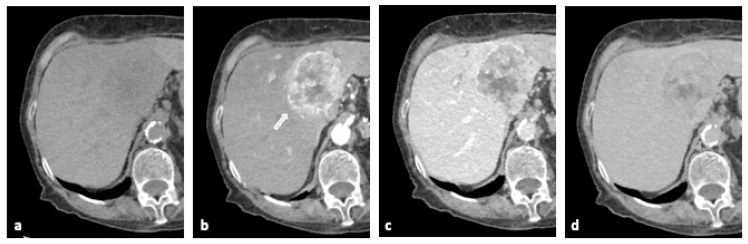
CE-CT features of iCCA. An example of the enhancement of iCCA from CE-CT: (**a**) unenhanced CT: hypodense mass. (**b**) The arterial phase shows peripheral border enhancement (arrow). (**c**) The portal venous phase and (**d**) the delayed phase show progressive centripetal enhancement. Abbreviations: iCCA, intrahepatic cholangiocarcinoma; CE-CT, contrast-enhanced computed tomography; CT, computed tomography.

Mass-forming iCCA appears on unenhanced CT images as iso-hypodense when compared to the surrounding healthy hepatic parenchyma; this characteristic is determined by the hypovascular nature of the tumor [31]. After the administration of the contrast medium, the lesion shows an enhancement of the peripheral rim of the lesion in the arterial phase, with progressive centripetal enhancement in the subsequent phases and typically delayed enhancement [25] (Figure 2). Satellite nodules may also be associated [25].

The pattern of enhancement after the injection of a contrast medium reflects the histological characteristics of the tumor: the hyper-density of the peripheral border in the arterial phase which disappears in the venous phase is due to the presence of high-growth tumor cells in this area.

Instead, delayed enhancement is mainly caused by the presence of fibrotic tissue occupying the central area of the lesion [31]. The presence of this tissue can lead to the retraction of the liver capsule, a finding that is present in about a third of tumors [26]. In some cases, delayed enhancement may not be present if the tumor has necrosis or cells containing mucin [21].

More frequently, pCCA and dCCa have the morphology of periductal-infiltrating tumors [26]. The tumor presents as an abruptly terminating lesion or an asymmetrical thickening of the wall of the affected bile duct. The involved bile ducts show concentric thickening of the walls, appearing as a ring or a prominent spot [33].

Intraductal-growing cholangiocarcinoma represents a group of tumors that manifests as a thickening of the wall of the bile duct or a mass growing into the lumen [33]. Various types of characteristics can be identified, such as marked ectasia of the bile ducts in the presence or absence of a visible mass, lesions within a dilated duct, or a mass within a focal dilatation of the duct [25].

### 2.3. Magnetic Resonance Imaging (MRI)

Nowadays, the method of choice for the diagnosis and staging of CCA is MRI.

In the diagnosis of iCCAs less than 5 cm in size, MRI has a sensitivity and specificity of 77% and 96%, respectively; while in diagnoses greater than 5 cm, it has a sensitivity of 53% and a specificity of 95% [27]. For pCCA, MRI has a sensitivity of about 89% in patients with primary sclerosing cholangitis, and this drops to 71% in asymptomatic patients [34]. The proximal bile ducts are evaluated through magnetic resonance cholangiopancreatography (MRCP), which detects bile duct involvement in 71–96% of cases [29].

The classic method of study for CCA sees the use of T1- and T2-weighted sequences, diffusion-weighted imaging (DWI), and dynamic contrast sequences (DCE-MRI), including an hepatobiliary phase in case of hepatospecific MRI contrast agents [21]. Examination can be completed with T2-weighted cholangiography (MRCP) sequences to map the biliary tree [32].

**Figure 3:**
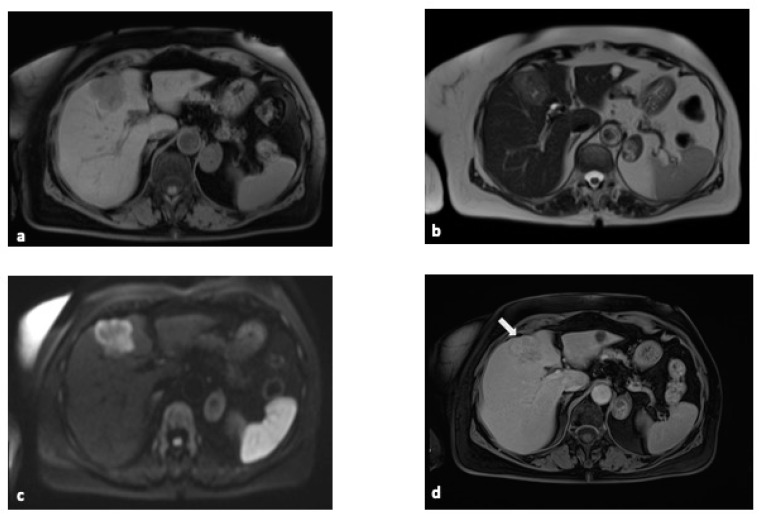
MRI features of iCCA. MRI features in a 77-year-old female with a diagnosis of intrahepatic cholangiocarcinoma: (**a**) hypointense on the T1-weighted sequence; (**b**) hyperintense on the T2-weighted sequence; (**c**) target appearance in DWI images: peripheral hyperintensity and central hypointensity; and (**d**) progressive enhancement in DCE-MRI images. A retraction of the hepatic capsule can also be observed (arrow). Abbreviations: MRI, magnetic resonance imaging; DWI, diffusion-weighted imaging; DCE-MRI, dynamic contrast sequences.

Mass-forming iCCA presents as hypo- or isointense lesions on T1-weighted sequences, while it appears as hyperintense lesions on T2-weighted sequences according to the amount of stroma and mucin in the lesion [18] (Figure 3).

Dynamic acquisitions following gadolinium administration in MRI demonstrate enhancement patterns similar to those observed with CT, providing crucial diagnostic insights. The most common pattern is a rim of peripheral enhancement during the arterial phase, which progresses to more pronounced enhancement in the delayed phase [18]. In contrast, smaller lesions with a lower fibrotic component exhibit intense enhancement in the arterial phase but less prolonged enhancement in the delayed phase [32]. Additional imaging features that aid in diagnosis include capsular retraction, vascular encasement, and the presence of satellite nodules [35], which collectively contribute to the accurate characterization of cholangiocarcinoma.

In high b-value DWI images, iCCA exhibits a characteristic target appearance (Figure 3c) due to peripheral hyperintensity and central hypointensity. Peripheral hypointensity and central hyperintensity are highlighted on the apparent diffusion coefficient (ADC) map; these features show that in the peripheral portion, there is a high cellular component, while in the central portion, the fibrotic component prevails [16].

Tumor cells have an epithelial origin and therefore are not sensitive to hepatocyte-specific MRI contrast agents. After their administration, the lesion remains hypoenhanced with respect to the surrounding parenchyma, allowing for better visualization of the margins of the lesion [26]. However, some studies show a different uptake of hepatospecific contrast agents by iCCA, which appears irregularly hypointense due to the presence of fibrotic stromal tissue; consequently, the hepatospecific contrast agent can accumulate in this area, showing a hyperintense image [36]. Therefore, the lesion appears hypointense in the periphery and centrally hyperintense [35].

Periductal-growing CCA appears on T2-weighted and MRCP sequences as lumen thickening and narrowing of the affected bile duct associated with the dilatation of the proximal intrahepatic bile ducts. After contrast administration, it usually shows delayed enhancement [21].

Periductal-growing CCA represents approximately 70% of all pCCA cases. During MRI, it appears as long biliary stenoses, characterized by wall thickening and enhancement in the arterial and portal phases; in some cases, there may be the infiltration of periductal fat [37].

Intraductal papillary tumors are identified by MR cholangiography, which allows for analysis of the biliary tree [33]. Intraductal CCA shows a T2 hyperintense and T1 hypointense signal [18]. It shows heterogeneous enhancement in the early phase and improves in the delayed phases, such as in mass-forming CCA [35].

A more in-depth comparative analysis of the various imaging modalities is presented in Table 2.

## 3. Radiomics and Artificial Intelligence in Medical Imaging

Radiological images possess numerous data and quantitative characteristics that the human eye is unable to identify. Radiomics plays a key role in the analysis of these features which provide information on the shape, size, volume, and consistency of the neoplasm. Consequently, radiomics allows for a quantitative analysis of radiological images by extracting data that are associated with clinical objectives [38]. The mechanism behind this emerging non-invasive tool is the imaging manifestation of molecular pathological processes that are not detected by simple image analysis [39].

Radiomics is a multi-step process that permits the extrapolation of quantitative and characteristic data from radiological images through the use of artificial intelligence (AI), which significantly improves the construction of predictive radiomic models through a better selection of radiomic characteristics [39,40].

**Figure 4:**
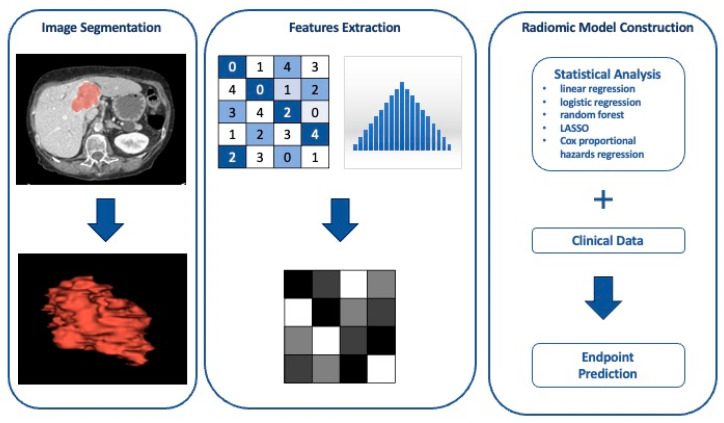
Radiomics workflow. Schematic representation of radiomics workflow. Abbreviations: LASSO, least absolute shrinkage, and selection operator.

The radiomics workflow has several stages (Figure 4). The first phase of the radiomic process is the acquisition of images, from which features can be extracted through a segmentation process. The entire lesion is circumscribed by tracing regions of interest (ROIs), taking care to avoid unnecessary tissue portions that could distort feature extraction [40].

More frequently, segmentation is performed through manual or semi-automatic methods through the use of segmentation algorithms such as region-growing or thresholding algorithms, which are then manually adjusted. Nowadays, segmentation algorithms based on the use of deep learning are emerging, which allow for automatic segmentation of the region of interest [41].

Once the segmentation process is completed, the quantitative features of the lesion are extracted. Various types of features can be identified: shape features contain the dimensional properties of the lesion; first-order statistical characteristics describe the intensity of the single voxel; second-order statistical features describe the heterogeneity of the lesion through information on the arrangement of individual voxels; and higher order statistical characteristics are obtained through statistical analyzes and through the application of filters or mathematical algorithms [42].

A fundamental process in the creation of the radiomic model is the selection of features. First of all, a final endpoint must be identified, from which the feature selection process will start. Through specific unsupervised approaches, only the most appropriate characteristics are selected with respect to the pre-established endpoint [40]. Among the unsupervised approaches, the most frequently applied are cluster analysis followed by principal component analysis: the first method involves the creation of groups of characteristics with high intra-cluster redundancy and low correlation, from which a single characteristic is selected and used for the association analysis; the second method is used to explain the variability among extracted features through the use of a small group of unrelated features identified among a large number of related features. Consequently, the chosen characteristics can be used for association analysis [42].

The final step of the radiomic process consists of the construction of a mathematical model through supervised multivariate analysis in order to correlate the extracted characteristics with the clinical data to achieve the pre-set result [42]. There are a number of statistical, machine learning, and data mining methods used to evaluate endpoints, including linear and logistic regression, random forest, Cox proportional hazards regression, decision tree, least absolute shrinkage and selection operator (LASSO), support vector machines (SVM), deep learning, and neural network learning [40].

Radiomics is a constantly evolving field and has been shown considerable interest in clinical studies for diagnostic and prognostic purposes; however, it has limitations (Table 3). First, the lack of standardization of the image acquisition and reconstruction process makes the studies difficult to reproduce due to their retrospective nature [41]. An interesting method to eliminate image acquisition bias could be the use of an automatic acquisition protocol, with encouraging results in terms of achieving robustness of radiomic parameters. In this way, through a test–retest analysis, it is possible to eliminate the radiomic characteristics that present greater variability [40]. The segmentation process can also generate variations in the analysis of radiomic characteristics. The manual segmentation process is hugely time-consuming and depends on the skill of the radiologist; therefore, there is high variability in results. On the other hand, the automatic and semi-automatic methods have demonstrated success only in homogeneous lesions, with a lower degree of variability [40].

Nowadays, the research field regarding radiomics is increasingly evolving, making the radiomics process a potential tool to assist the clinical management of patients [15].

There are many studies in the literature that demonstrate how the use of radiomics can improve the diagnosis and treatment of cancer [43]. A study conducted by Wu et al. demonstrated the existence of 53 radiological features capable of predicting a type of non-small cell lung cancer [44]. A radiomics model has also been applied for the management of patients with colon cancer, proving able to predict disease-free survival [45], defined as the length of time after treatment during which the patient remains free from any signs or symptoms of the cancer [46]. Again, radiomic features extracted from non-contrast magnetic resonance images can be used to distinguish the degree of differentiation of HCC [47].

The radiomic process of extracting and selecting features leverages AI mechanisms, particularly machine learning, to identify the most significant characteristics [48]. AI, a field of engineering research focused on addressing complex problems, employs innovative solutions such as artificial neural networks [40,49]. These neural networks play a crucial role in enhancing the radiomic workflow by enabling the efficient analysis and interpretation of vast datasets, ultimately improving diagnostic accuracy and decision-making in clinical practice.

AI in medicine is applied in two distinct areas: in the physical field, which sees the use of AI in the development of medical devices and technological tools that help in clinical practice; and in the virtual field using machine learning, a particular algorithm that improves learning through experience [49]. Machine learning is divided into supervised learning, where the algorithm is supplied with data that are then used for its development, and unsupervised learning, where the system must autonomously classify the supplied data [50].

Nowadays, deep learning (DL) is attracting increasing interest in various areas of the scientific community, in particular, a branch of DL called machine learning, capable of training to recognize the differences between various characteristics in order to approximate very complex relationships [13]. DL algorithms are based on the use of convolutional neural networks (CNNs), which are complex systems capable of merging many inputs of information arriving from various levels, in a similar way to the human neuronal system. Each neural network can combine various inputs that come from lower levels in order to generate a much more complex output. DL involves a neural network characterized by a high number of these levels, thus allowing for enormous computing power [51].

Recently, the interest in AI in the field of radiology is gaining ground. Algorithms have been developed, called computer-aided detection (CAD), that can help radiologists understand images [13]. CAD takes advantage of DL to create models that are able to classify the disease and to discriminate the degree of malignancy, as well as to predict eventual tumor development [52]. These systems are widely used in the breast cancer field, with an accuracy similar to that of radiologists in the detection of breast cancer, or superior with regard to microcalcifications [52]. CAD systems have been developed that can identify lung nodules on CT images and prostate cancer on magnetic resonance imaging. However, the use of CAD in clinical settings remains questionable, as very often they do not facilitate the radiologist’s work; on the contrary, they make it more laborious as they have to evaluate each result [13].

There are AI systems that are able to reduce the artifacts generated by image acquisitions with low radiation levels: in this way, diagnostic images can be produced even at lower doses of radiation, making examinations such as CT or Positron Emission Computerized Tomography (PET-CT) more accessible. Even magnetic resonance can be improved with the use of AI; in particular, deep learning allows for reductions in the acquisition time of sequences, improving the quality of the exam [50].

AI makes it possible to evaluate the quantitative characteristics of a lesion, extrapolating from these imaging markers that could be used to predict the likelihood of malignancy, prognosis, and response to treatment. Deep learning can also be applied to disease monitoring through a data comparison protocol that is used to quantify lesion changes [13].

Many artificial intelligence algorithms exceed human performance, but it will still be impossible to replace the role of radiologists, who will always have to validate and supervise this process [13]. Large studies are needed to test the real performance of artificial intelligence mechanisms compared to human intelligence, which require high costs and timescales [51]. There are also medico-legal consequences regarding privacy and data security [14].

Radiomics and artificial intelligence can be of great help in the diagnosis and management of patients with CCA; therefore, the purpose of this review and of the following paragraph is to examine, among the various studies in the literature, how these two mechanisms can influence the path therapeutic diagnosis of CCA.

## 4. Radiomics and Artificial Intelligence Applications in Cholangiocarcinoma

Even if the role of radiomics in the evaluation of patients with CCA is yet to be established during routine imaging detection, the use of radiomics and artificial intelligence keeps increasing in this field.

This review depicts how the radiological features of CCA can be analyzed through radiomics and with the help of AI for many different purposes such as the following: differential diagnosis, the prediction of lymph node metastasis, the defining of prognostic groups, and the prediction of early recurrence (Table 4).

### 4.1. Differential Diagnosis

Histology remains the reference standard in diagnosing CCA, and many studies have tried to develop models to differentiate hepatocellular carcinoma from other lesions through radiomics features and artificial intelligence. Hanyue Xu et al. [57] explored the possibility of combining texture parameters with machine learning methods to distinguish iCCA and hepatic lymphoma in contrast-enhanced computer tomography. They built 45 predictive models, with encouraging results because most of the models showed good performance, with a large area under the curve (AUC > 0.85) and high accuracy (>0.85).

In another study, Rong Hu et al. [54] exploited an automated tree-based optimization tool programmed for the genetic differentiation of HCC from iCCA by applying multiparametric MRI. Both manual and automated analyses were performed to select an optimal ML model. The results showed an accuracy of 73–75% (95% CI 0.59–0.85) for the manual approach (sensitivity: 70–75% and specificity: 71–79%), while automated ML showed an accuracy of 73–75% (sensitivity: 65–75% and specificity: 75–79%). The results highlighted how the automated ML performance was similar to the manual optimization performance, and it could classify HCC and iCCA with a sensitivity and specificity comparable to that of radiologists. Improvements in the differentiation between iCCA and poorly differentiated HCC were reached by Chen X. and colleagues; they demonstrated how both radiomics and clinical-radiomics models achieved an AUC of 0.86 and 0.89 in a cohort of 134 patients who underwent Gadoxetic acid-enhanced MRI. Furthermore, the radiomics features selected for the clinical-radiomics model obtained from the random forest algorithm showed high net clinical benefit [62].

In a recent study, Xiaoliang Xu et al. [55] tried to classify HCC and iCCA based on radiomic analysis through a retrospective study. They developed a radiomic-based model in a training cohort of 122 patients and extracted radiomic features from CT scans. The training set showed an AUC of 0.855 for radiomics, compared with 0.689 for radiologists. In the valuation cohorts, the AUC of the evaluation was 0.847 and the validation was 0.659, which indicated that the established model had a significantly better performance in distinguishing HCC from CCA. Also, other model were built by different research groups with accuracy, higher than 88% for TC in a cohort of more than 4000 patients with different liver lesions [63], to 96% in a cohort of 814 patients with CCA, HCC, and liver metastasis [64].

### 4.2. Pre-Treatment Assessment

Correct pre-treatment staging is essential to tailor therapeutic approaches to each patient, and a few studies have tried to reach this goal by using radiomics and AI to identify the primary lesion and to assess lymphatic node metastasis, vascular invasion, and perineural invasion in CCA [65,66].

#### 4.2.1. Identification and Segmentation of Primary Lesion

Identifying and segmenting extrahepatic CCA from medical imaging can be challenging, and AI can support this process. Yang C. at al. [67] tested their hypothesis in a retrospective cohort of 137 patients examined with MRI (T2w, T1w, and DWI imaging). The 3D VB-Net system was used to build different models based on the different sequences acquired, and this was compared with manual segmentation, which was considered to be ground truth. The DWI model showed the best dice similarity coefficient compared to the ground truth of 0.786, with a success rate of identification and segmentation of 0.98, 0.78, and 0.72 in the training, testing, and validation subsets, respectively.

#### 4.2.2. Lymphatic Node Metastasis (LNM)

Radiomics has been tested as a potential tool to assess metastatic nodes in the pre-operative setting, with promising results; for example, Yong Tang et al. [58] made an analysis through machine learning-based radiomics to predict lymphatic node metastasis of eCCA on a group of 100 patients diagnosed with eCCA confirmed by pathology. A total of 1200 radiomics features were extracted from axial T1-weighted imaging (T1WI), T2-weighted imaging (T2WI), DWI, and ADC images. A systematical framework considering combinations of five feature selection methods and ten machine learning classification algorithms (classifiers) was developed and investigated. For LNM prediction, the feature selection method minimum redundancy maximum relevance (mRMR) and classifier eXtreme Gradient Boosting achieved the best performance (AUPRC = 0.95, AUC = 0.98 (95% CI 0.94–1.00), ACC = 0.90 (95% CI 0.77–1.00), sensitivity = 0.75 (95% CI 0.30–0.95), and specificity = 0.94 (95% CI 0.72–0.99))

In a previous paper, Lei Xu et al. [61] used radiomics with the support vector machine (SVM) approach applied on MRI to assess preoperative lymph node status in iCCA. One-hundred and six iCCAs were used to train the model, and image features were selected from post-contrast T1-weighted images. An SVM score was obtained for each iCCA case to reflect the LN malignancy status probability. Then, a combination nomogram was built, merging the SVM score and clinical features. For the validation cohort, another 42 iCCAs were used. The SVM score showed the ability to significantly discriminate between LNs with and without metastases in both groups (the training group: 0.5466 vs. 0.3226, *p* < 0.0001; the validation group: 0.5831 vs. 0.3101, *p* = 0.0015). The nomogram, incorporating the SVM score, the CA 19-9 level, and the MR, provided an individualized LN status evaluation and helped clinicians guide their surgical decisions.

#### 4.2.3. Vascular Invasion

Microvascular invasion (MVI) represents an important prognostic factor, in particular in preoperative setting, and helps the decisional process for the establishment of adjuvant or neo-adjuvant treatment strategies and individualized surveillance programs.

In a multicentric retrospective study, Wenyu Gao et al. [56] developed a multiparametric fusion deep learning model based on DCE-MRI to predict microvascular invasion in I-CCA in a preoperative setting. A total of 519 patients with a single iCCA were divided into training, validation, and an external test groups (n = 361, 90 and 68, respectively), and DL models of a multiparametric fusion CNN and a late fusion CNN were constructed to evaluate MVI in iCCA. In the external test group, the proposed model with the multiparametric fusion CNN achieved an AUC of 0.888 (accuracy, sensitivity, and specificity; 86.8%, 85.7%, and 87.0%, respectively) in the assessment of MVI in iCCA. On the other hand, the model with late fusion DL achieved a slightly lower AUC of 0.866 (accuracy, sensitivity, and specificity: 83.8%, 78.6%, and 85.2%, respectively) for evaluating MVI in iCCA.

#### 4.2.4. Neural Invasion

Although only a few authors have performed studies on the topic, perineural invasion in CCA represents an important independent risk factor of recurrence and survival. Liu Z. and colleagues [68] developed a retrospective and prospective study including 243 patients, who underwent preoperative CT analyzed with radiomics to build a signature with machine learning which was able to discriminate between the presence or lack of presence of perineural invasion. The results showed a signature composed by seven radiomic features with an AUC of 0.88, 0.83 and, 0.83 in the training, retrospective external validation, and retrospective external validation groups, respectively. Furthermore, survival analysis performed on the combined model resulted in the ability to stratify patients and a hazard ratio of 1.933.

### 4.3. Prognostic Prediction

Due to the underhanded onset and rapid progression of iCCA, most patients receive a diagnosis at advanced stages, with only 30 to 60% of iCCA patients eligible for surgery with curative intent. Even the postoperative prognosis of patients who undergo “curative-intent” hepatectomy is poor, and radiochemotherapy provides a modest survival benefit for unresectable cases. Therefore, the possibility of developing a robust prognostic prediction tool for iCCA would represent great clinical value for treatment and strategy choices.

Youyin Tang et al. [59] studied the preoperative prognostic value of a CT radiomics nomogram combined with ML in patients with iCCA through a retrospective study involving 101 patients with pathological confirmation of intrahepatic cholangiocarcinoma. The authors developed a radiomics nomogram by including both the radiomics score and independent clinical risk factors selected from multivariate analysis, also assessed by the calibration curve, the ROC curve, and the survival curve. Multivariate Cox analysis showed three independent prognostic factors. The radiomics nomogram showed significant prognostic value and overall survival with differences in the 1-year vs. 3-year survival rates of stratified high-risk and low-risk patients (30.4% vs. 56.4% and 13.0% vs. 30.6%, respectively, *p* = 0.018), with potential clinical application in the preoperative setting.

### 4.4. The Prediction of Early Recurrence

Up to 40–65% of patients with pCCA rapidly progress to early recurrence (ER) even after curative resection, therefore, the creation of a reliable prognostic system has become paramount for quantifying the risk of ER to determine post-operative strategies and facilitate personalized management. In a recent study, Huan Qin et al. [60] used machine learning radiomics to predict early recurrence in perihilar cholangiocarcinoma after curative resection. With a study cohort of 641 patients with pathologically confirmed pCCA who underwent surgical resection, they developed a multilevel model integrating multi-omics and machine learning-based radiomics algorithms to predict the ER of pCCA after curative resection.

A machine learning analysis of 18,120 radiomic features based on multiphase CECT and 48 clinico-radiologic characteristics was performed for a multilevel model with seven independent factors, which quantified the risk of ER. With an area under the curve (AUC) of 0.883, the model showed a higher accuracy than those of conventional staging systems (AJCC 8th TNM staging system AUC 0.641).

In another study, Yangda Song et al. [53] tried to validate a preoperative model for predicting ER risk after curative resection of intrahepatic cholangiocarcinoma iCCA through an AI-based CT radiomics approach in a cohort of 311 patients from eight medical centers who underwent curative resection.

A combined clinical–radiomics model that included 15 radiomic features and three clinical features (CA19-9 > 1000 U/mL, vascular invasion and tumor margin), resulted in areas under the curve (AUCs) of 0.974 (95% CI 0.946–1.000) in the derivation cohort, and 0.871–0.882 (95% CI 0.672–0.962) in the internal and external validation cohorts, respectively, which are higher than the AJCC 8th TNM staging system (AUCs: 0.686–0.717, *p* all < 0.05).

As shown by several studies [69,70], some of which are reported above, AI-driven combined radiomics model may become a useful tool in predicting ER.

### 4.5. Palliative Measures

Palliative measures like stent placement, recommended for inoperable hilar CCAs, may also be analyzed by AI models. For example, Shao et al. [71] developed an artificial neural network (BP-ANN) model to predict early occlusion of bilateral plastic stents for inoperable hilar cholangiocarcinoma (pCCA) in a cohort of 288 patients. Multivariate analysis showed that the cancer stage and the Bismuth stage were independently and significantly associated with early stent occlusion (*p* = 0.005 and *p* = 0.003, respectively). The training cohort BP-ANN showed a larger AUC than the multivariate logistic regression model (*p* = 0.00049), and the internal testing cohort confirmed the result (*p* = 0.02142).

## 5. Future Directions

This review of the literature highlights a wide range of studies utilizing radiomics and AI to evaluate CCA, while also revealing numerous topics and approaches that remain underexplored. One particularly intriguing avenue for future research is the potential to identify precursors of CCA by analyzing the liver parenchyma before a lesion becomes detectable to the human eye. Such advancements could pave the way for early, personalized treatment strategies, significantly improving patient outcomes and altering the clinical trajectory of this challenging disease. Hopefully, we will soon be able to develop standardized radiomics models and protocols to diagnose lesions as being CCA without any delay, and also give information on the prognosis to the patient. Furthermore, interesting results are emerging in the multidisciplinary approach, including, for example, the added value of AI, and in particular of natural language processing, in the managing of multidisciplinary meetings for therapeutic decisions in patients with CCA [72].

To advance the field, it is essential for future studies to be prospective, multicentered, and conducted on large patient cohorts to validate the role of radiomics and AI in the diagnosis, treatment, and prognosis of CCA, as well as their practical effectiveness in clinical settings. The generalizability of current findings remains limited due to the retrospective nature of most existing data and the small patient cohorts typically analyzed in published studies. Addressing these limitations through well-designed, multicentered prospective trials would provide more robust and reliable evidence. Such efforts would strengthen the clinical value of these emerging technologies, ensuring that their potential benefits can be effectively translated into routine medical practice.

## 6. Conclusions

As of now, CCA still has an extremely unfavorable prognosis. Scientific research, with its novelty in quantitative imaging combined with AI, can lead to valuable improvements in the early detection, characterization, and pre-treatment staging of these tumors, and therefore guide clinicians to undertake optimal therapeutic strategies.

We expect soon the wide application of radiomics and AI in the diagnosis of CCA and in many other radiological fields; therefore, we recommend that healthcare professionals are educated and prepared for the ever-increasing role of radiomics in the diagnosis, treatment, and prognosis of CCA, and at the same time are informed on their current limits.

As with all forms of technological advancement, the adoption of radiomics and AI in clinical practice faces numerous and complex limitations and barriers. These challenges range from the standardization of imaging techniques and the validation of methodologies to the seamless integration of these emerging technologies into hospital workflows. Additionally, ethical concerns related to AI implementation must be carefully addressed to ensure responsible and transparent clinical use.

In conclusion, radiomics and AI could have the potential to overcome the limitations of subjective imaging evaluation by providing objective data on lesions, such as their histology and staging, and could play a crucial role in predicting patient survival. Physicians should see radiomics and AI as essential imaging tools in the evolving era of patient-centered precision therapy.

## Figures and Tables

**Table 1:** Bismuth–Corlette classification.

Type	Description
Type I	Below the confluence of the hepatics duct
Type II	Involves the confluence but not the hepatic ducts
Type IIIa	Affects the common hepatic duct and the right hepatic duct
Type IIIb	Affects the common hepatic duct and the left hepatic duct
Type IV	A multicentric tumor, or extended to the confluence of the right and left hepatic ducts

**Table 2:** Comparison of imaging modalities for diagnosing and staging cholangiocarcinoma.

Imaging Modality	Strengths	Limitation	Sensitivity	Specificity	PPV	NPV
Ultrasound (US)	Widely available, non-invasive, and cost-effective.	Operator-dependent, limited in obese patients, and less effective for deep lesions.	60–80%	50–75%	60–70%	55–75%
Computed Tomography (CT)	High spatial resolution, good for staging and assessing vascular invasion.	Radiation exposure and limited soft tissue contrast.	80–90%	70–85%	75–85%	80–90%
Magnetic Resonance (MR)	Excellent soft tissue contrast, superior for biliary tract evaluation.	High cost, longer exam time, and limited availability.	85–95%	80–90%	85–90%	85–95%

A summary table highlighting the advantages and weaknesses of each imaging modality used for diagnosing and staging cholangiocarcinoma. Abbreviations: US, ultrasound; CT, computed tomography; MR, magnetic resonance; PPV, positive predictive value; NPV, negative predictive value.

**Table 3:** Key applications of radiomics and AI in medical imaging.

	Description	Challenges/Limitations
AI in Medical Imaging	AI uses neural networks to solve complex problems in imaging.	Limited clinical adoption due to regulatory and legal issues.
Machine Learning	Supervised and unsupervised learning classify and predict data.	Data quality and diversity impact model performance.
Deep Learning (DL)	CNNs process and interpret imaging data.	High computational demands and data requirements.
CAD Systems	Assist radiologists, especially in cancer detection.	May increase workload due to false positives.
Image Enhancement	Reduces image artifacts, improves quality at lower radiation doses.	Integration with existing systems is still evolving.
Predictive Analytics	AI-based markers predict malignancy, prognosis, and treatment response.	Requires extensive validation and clinical trials.

A table summarizing the main applications of radiomics and AI in medical imaging. Abbreviation: CNN, convoluted neural network; AI, artificial intelligence.

**Table 4:** Applications of AI in CCA.

Study	*N* Patients	Endpoint	Type of Evaluation	Model Performance	Imaging Modality	Data Augmentation	Processing Steps	Features Extracted	Nature of Study
Yangda Song et al. (2023) [53]	Total 311	Prediction of early recurrence of I-CCA	AI and radiomics	End 1: AUC 0.974	CT	No	LightGBM model	15	Multicentric Retrospective
Rong Hu et al. (2022) [54]	Total 489	Differentiation of HCC from I-CCA	Automated machine learning	End 1: AUC 0.89	MRI	No	AutoML	94	Monocentric Retrospective
Xiaoliang Xu et al (2022) [55]	Total 211	Classification of HCC and I-CCA	Radiomic Analysis	End 1: AUC 0.855	CT	No	SVM	841	Monocentric Retrospective
Wenyu Gao et al. (2022) [56]	Total 519	Prediction of Microvascular Invasion in I-CCA	Fusion Deep Learning	End 1: AUC 0.888	DCE-MRI	No	CNN		Monocentric Retrospective
Hanyue Xu et al. (2021) [57]	Total 129	Distinguish I-CCA and HL	Radiomics and Machine Learning	End 1: AUC 0.997	CECT	No	ML model	38	Monocentric Retrospective
Yong Tang et al. (2021) [58]	Total 100	Preoperative prediction of differentiation degree and lymph node metastasis in E-CCA	Radiomics and Machine Learning	End 1: AUC 0.9	MRI	No	BACG JMI	1200	Monocentric Retrospective
Youyin Tang et al. (2021) [59]	Total 101	Preoperative prognostic value in I-CCA	Radiomics nomogram and Machine Learning	End 1: AUC 0.783 End 2: AUC 0.633	CT	No	SVM	42	Monocentric Retrospective
Huan Qin et al. (2020) [60]	Total 274	Prediction of early recurrence in perihilar CCA after curative resection	Radiomics and Machine Learning	End 1: AUC 0.883	CECT	No	A-Shrink, V-Shrink, P-Shrink	302	Monocentric Retrospective
Lei Xu et al. (2019) [61]	Total 42	Preoperative lymph node status evaluation in I-CCA	Radiomics approach based on support vector machine	End 1: AUC 0.842	MRI	No	SVM	5	Monocentric Retrospective

A table summarizing the main studies that have focused on applications of AI in the detection of CCA. Abbreviation: CCA, cholangiocarcinoma; I-CCA, intrahepatic cholangiocarcinoma; AI, artificial intelligence; HCC, hepatocellular carcinoma; CT, Computer Tomography; CECT, contrast-enhanced computer tomography; DCE-MRI, dynamic contrast-enhanced, HL, hepatic Lymphoma; E-CCA, Extrahepatic cholangiocarcinoma; CNN, convolutional neural network; ML, machine learning; BACG, Bagging Classifier; JMI, joint mutual information; SVM, support vector machine.

## Data Availability

No new data were created or analyzed in this study. Data sharing is not applicable to this article.
